# The role of retinol dehydrogenase 10 in the cone visual cycle

**DOI:** 10.1038/s41598-017-02549-8

**Published:** 2017-05-24

**Authors:** Yunlu Xue, Shinya Sato, David Razafsky, Bhubanananda Sahu, Susan Q. Shen, Chloe Potter, Lisa L. Sandell, Joseph C. Corbo, Krzysztof Palczewski, Akiko Maeda, Didier Hodzic, Vladimir J. Kefalov

**Affiliations:** 1 Department of Ophthalmology & Visual Sciences, Washington University School of Medicine, St. Louis, Missouri 63110 USA; 2 Department of Ophthalmology and Visual Sciences, Case Western Reserve University, Cleveland, Ohio 44106 USA; 3 Department of Pathology & Immunology, Washington University School of Medicine, St. Louis, Missouri 63110 USA; 4 Department of Oral Immunology and Infectious Diseases, University of Louisville, Louisville, Kentucky 40202 USA; 5 Department of Pharmacology and Cleveland Center for Membrane and Structural Biology, Case Western Reserve University, Cleveland, Ohio 44106 USA; 6 Department of Genetics, Harvard Medical School, Boston, MA 02115 USA; 7 MilliporeSigma, St. Louis, MO 63103 USA

## Abstract

Pigment regeneration is critical for the function of cone photoreceptors in bright and rapidly-changing light conditions. This process is facilitated by the recently-characterized retina visual cycle, in which Müller cells recycle spent all-*trans*-retinol visual chromophore back to 11-*cis*-retinol. This 11-*cis*-retinol is oxidized selectively in cones to the 11-*cis*-retinal used for pigment regeneration. However, the enzyme responsible for the oxidation of 11-*cis*-retinol remains unknown. Here, we sought to determine whether retinol dehydrogenase 10 (RDH10), upregulated in rod/cone hybrid retinas and expressed abundantly in Müller cells, is the enzyme that drives this reaction. We created mice lacking RDH10 either in cone photoreceptors, Müller cells, or the entire retina. *In vivo* electroretinography and transretinal recordings revealed normal cone photoresponses in all RDH10-deficient mouse lines. Notably, their cone-driven dark adaptation both *in vivo* and in isolated retina was unaffected, indicating that RDH10 is not required for the function of the retina visual cycle. We also generated transgenic mice expressing RDH10 ectopically in rod cells. However, rod dark adaptation was unaffected by the expression of RDH10 and transgenic rods were unable to use *cis*-retinol for pigment regeneration. We conclude that RDH10 is not the dominant retina 11-*cis*-RDH, leaving its primary function in the retina unknown.

## Introduction

Absorption of light by the chromophore of visual pigments in vertebrate photoreceptor cells results in its conversion from 11-*cis*- to all-*trans*-retinal. This isomerization activates the phototransduction enzymatic cascade and leads to the generation of a light response (reviewed in refs 1, 2). Resetting the activated visual pigment to its inactive ground state occurs by the release of all-*trans*-retinal and its recycling back to 11-*cis*-retinal in a process known as the visual or retinoid cycle (reviewed in ref. 3). For rod photoreceptors, the supply of recycled chromophore appears to be the rate-limiting step of visual pigment regeneration that controls the kinetics of dark adaptation^4–6^. This canonical visual cycle involves light-independent processing of the all-*trans*-chromophore by the retinal pigmented epithelium (RPE) followed by transfer of the recycled 11-*cis*-retinal to both rod and cone photoreceptors in the adjacent retina (reviewed in refs 3, 7 and 8).

A second, cone-specific visual cycle that involves the neural retina rather than the RPE also has been identified^9^. Here, Müller glial cells within the neural retina convert all-*trans-*retinal back to 11-*cis*-retinol for selective use in cone cells^10, 11^, where it is oxidized to the 11-*cis*-retinal chromophore needed for pigment regeneration^12–15^. This intraretinal visual cycle provides a selective, rapid, and efficient chromophore supply to cone cells to drive their rapid dark adaptation and continuous function under bright and rapidly-changing light conditions^7, 16, 17^.

Despite the clear functional significance of the retina visual cycle, some of its key molecular components remain unknown. A recent study suggested that dihydroceramide desaturase-1 (DES1) is the Müller cell isomerase responsible for converting all-*trans-*retinol back to 11-*cis*-retinol^18^. A second enzyme, the multifunctional *O*-acyltransferase (MFAT), is believed to participate in the esterification of 11-*cis*-retinol, facilitating its storage in Müller cells^19^. Finally, cellular retinaldehyde-binding protein (CRALBP) expressed in Müller cells was found to play an important role in regulating the delivery of chromophore from Müller cells to cones^20^. However, the enzyme of the retina visual cycle responsible for the oxidation of 11-*cis*-retinol in cone photoreceptors remains unknown. As only cones, not rods, can oxidize *cis*-retinol to the *cis*-retinal needed for pigment regeneration^12–15, 21^, this unidentified cone-specific 11-*cis*-RDH is likely to be important in regulating access to the retina visual cycle.

Unlike wild-type rods, hybrid rod photoreceptors in the *rd7* mouse retina can employ the typically cone-specific retina visual cycle^5^. Notably, these hybrid rods also can use 11-*cis*-retinol for pigment regeneration and dark adaptation, suggesting that *rd7* rods might express the unidentified cone-specific 11-*cis*-RDH. At the start of the current study, differential expression analysis of *rd7* and wild-type retinas by RNA-seq revealed upregulation of retinol dehydrogenase 10 (*Rdh10*) in the *rd7* retina. RDH10 is a 38 kDa short-chain dehydrogenase/reductase (SDR) family member, previously reported to be expressed in the RPE and Müller cells^22–24^. Notably, RDH10 is predisposed towards oxidation and can use *cis*-retinoids as substrate^25^, both attributes fitting the profile of the unknown enzyme that oxidizes 11-*cis*-retinol in cone cells. Recently, the selective deletion of RDH10 in the RPE was found to delay both regeneration of rod visual pigment and rod dark adaptation^26^, indicating a role for this enzyme in the canonical RPE visual cycle. However, the possible involvement of RDH in the retina visual cycle and its function in the retina have not been investigated.

In this study, we sought to determine whether RDH10 is necessary and sufficient for enabling photoreceptor cells to utilize the retina visual cycle. We carried out loss-of-function experiments with mice lacking RDH10 in their cone cells, Müller cells, or the entire retina, and gain-of-function experiments with mice expressing transgenic RDH10 in their rod cells, to examine the potential role of RDH10 in the intraretinal visual cycle and cone function.

## Results

### Expression of RDH10 in the mouse retina

A recent study showed that hybrid rods in the *rd7* mouse, which lacks the rod developmental transcription factor Nr2e3^27^, can use the retina visual cycle^5^. Equally important, this study demonstrated that unlike wild-type mouse rod cells, *rd7* rods can employ the presumptive product of the intraretinal visual cycle, 11-*cis*-retinol, for pigment regeneration. Because the form of retinoid used for pigment regeneration is 11-*cis*-retinal, these results suggest that hybrid *rd7* rods have gained the normally cone-specific ability to oxidize 11-*cis*-retinol to 11-*cis*-retinal. Thus, *rd7* rods represent a unique model to potentially identify the RDH enzyme that drives the oxidation of 11-*cis*-retinol in cones.

We first examined existing microarray data from *rd7* mice^27^ which failed to reveal a likely RDH candidate gene for the oxidation of 11-*cis*-retinol. We next performed RNA-seq analysis of wild-type and *rd7* retinas and identified 416 genes significantly upregulated in *rd7* retinas (Table 1S; GEO accession number GSE86442), with only one known RDH among them, RDH10, that was upregulated ~3-fold in *rd7* compared to wild-type retinas.

To facilitate the expression analyses and subsequent functional characterization of the role of RDH10 in cone pigment regeneration, we generated several mouse lines with altered RDH10 expression. To determine if RDH10 is necessary or sufficient for enabling photoreceptors to oxidize 11-*cis*-retinol and access the retina visual cycle, we used mice carrying a conditional *Rdh10* allele (*Rdh10* ^*flox/flox*^)^28^. By crossing *Rdh10* ^*flox/flox*^ mice with the cone-specific *Hrgp-Cre* mouse line^29^, we generated cone-specific RDH10 knockout mice. Similarly, the *Pdgfra-Cre* line^30^ and the *Six3-Cre* line^31^ were used to knock out RDH10 in Müller cells and the entire retina, respectively. Finally, to investigate if RDH10 is sufficient for photoreceptors to use the retina visual cycle, we also generated transgenic mice that express RDH10 in rods (*Rdh10* ^+^ mice).

**Figure 1:**
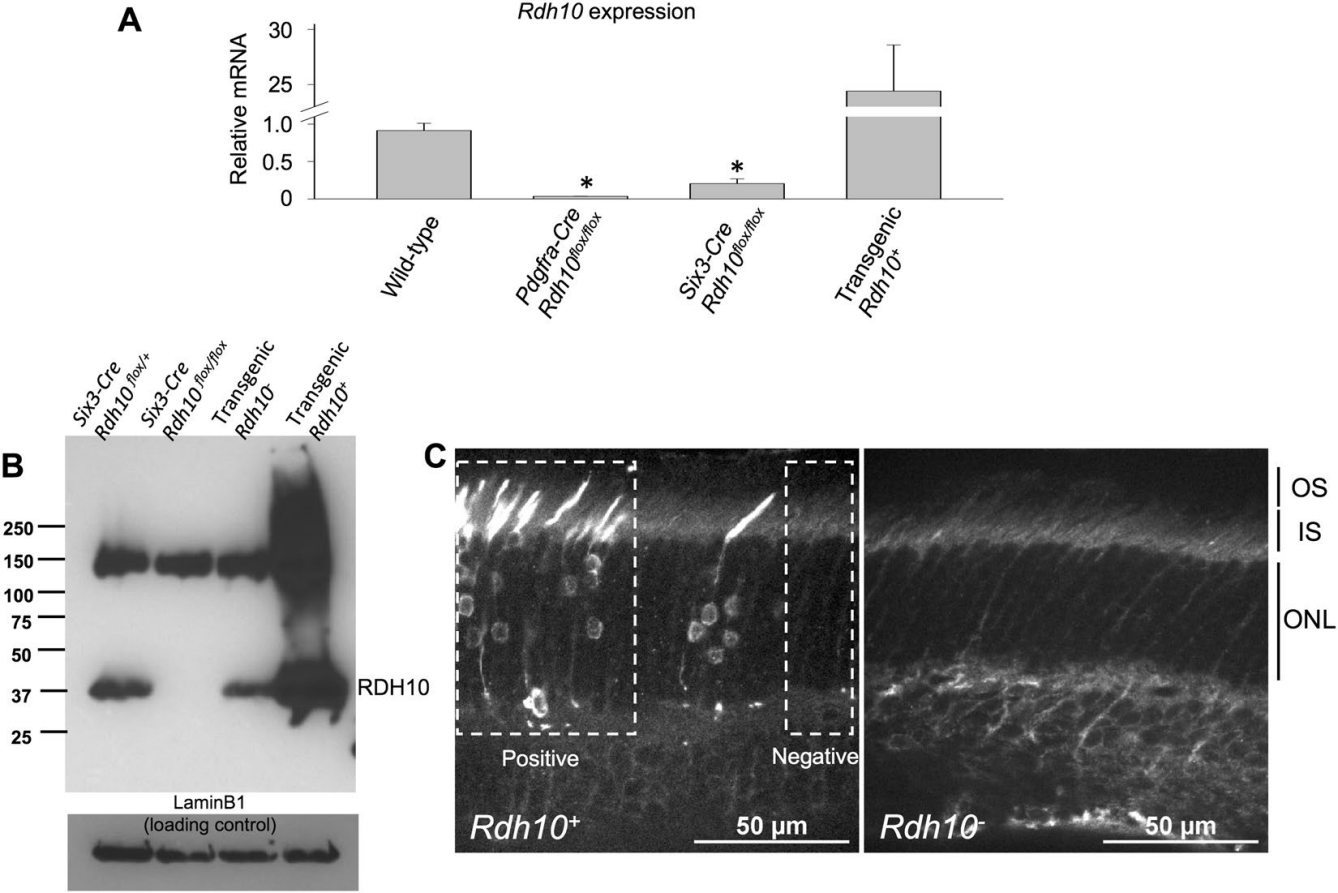
RDH10 expression in different mouse lines. (**A**) mRNA analysis of *Rdh10* expression in wild-type, Müller cell-specific RDH10 knockout (*Pdgfra-Cre Rdh10* ^*flox/flox*^), retina-specific RDH10 knockout (*Six-Cre Rdh10* ^*flox/flox*^), and RDH10 transgenic (*Rdh10* ^+^) mice. Data here and in all subsequent figures are shown as mean ± SEM. (**B**) Immunoblotting of *Six3-Cre* control, *Six3-Cre Rdh10* ^*flox/flox*^, transgenic *Rdh10* ^*−*^ (wild-type), and transgenic *Rdh10* ^+^ retinas. (**C**) Retinal section from a transgenic *Rdh10* ^+^ (left) and *Rdh10* ^*−*^ (wild-type) mice. The mosaic RDH10 expression in rods is shown in white and RDH10-positive and –negative sections are delineated by a box. OS, outer segment layer; IS, inner segment layer; ONL, outer nuclear layer.

We began by examining the level of *Rdh10* mRNA by RT-PCR. *Rdh10* mRNA levels were substantially reduced in retinas obtained from *Pdgfra-Cre Rdh10* ^*flox/flox*^ mice (Fig. 1A, left), indicative of its knockout in Müller cells. Similarly, the expression of *Rdh10* was also dramatically reduced in *Six3-Cre Rdh10* ^*flox/flox*^ retinas (Fig. 1A, center), demonstrating its suppression in the entire retina. In contrast, *Rdh10* mRNA levels were notably increased in transgenic *Rdh10* ^+^ mice, as expected (Fig. 1A, right). Due to the small number of cone cells in the mouse retina and the expected subtle at best change in *Rdh10* expression upon its selective deletion in cones, RT-PCR analysis of *Hrgp-Cre Rdh10* ^*flox/flox*^ retinas would not be informative and was not performed. However, the effective reduction of *Rdh10* mRNA in *Six3-Cre Rdh10* ^*flox/flox*^ mice demonstrates the sensitivity of the *Rdh10* ^*flox*^ allele to Cre excision in the retina. This, combined with the demonstrated lineage labeling of cones by the *Hrgp-Cre* driver^29^, would be expected to effectively eliminate expression of *Rdh10* from cone cells in retinas of *Hrgp-Cre Rdh10* ^*flox/flox*^ mice.

We also examined the expression of RDH10 protein in the retina by using a commercial RDH10 antibody. In agreement with the predicted molecular weight of RDH10 (NP_598593.1), an ~37 kDa protein was detected in lysates from wild-type retinas but was absent from *Six3-Cre Rdh10* ^*flox/flox*^ retinal lysates (Fig. 1B, left). As expected, RDH10 protein levels were markedly increased in transgenic *Rdh10* ^+^ retinas (Fig. 1B, right). Immunofluorescence microscopy of transgenic *Rdh10* ^+^ retinas revealed a mosaic pattern of RDH10 expression (Fig. 1C, left). In positive rods, transgenic RDH10 was detected in cell bodies along with potential accumulation in inner/outer segments and spherules. In contrast, no RDH10 expression was detected in sections of transgenic retinas with negative rods (Fig. 1C, left) and in rods from control retinas (Fig. 1C, right). Together, these results validate these transgenic animals as models for studying the effects of both loss- and gain- of RDH10 function in the retina visual cycle.

Our RDH10 immunoblot analysis also revealed an immunoreactive band with an apparent molecular weight of 150 kDa (Fig. 1B). Even though this protein was upregulated in *Rdh10* ^+^ retina, it was not downregulated in *Six3-Cre Rdh10* ^*flox/flox*^ retinas, suggesting that it corresponds to a cross-reaction of the RDH10 antibody. Accordingly, this band was later identified as Kinectin1 by mass spectrometry (data not shown). Although the reason for the upregulation of this protein in *Rdh10* ^+^ retinas remains unclear, this lack of antibody specificity is likely the reason for our inability to unambiguously visualize the expression of endogenous RDH10 in cones from retinal cross-sections (not shown).

### Cone RDH10 is not required for the normal function of dark-adapted cones

We began our physiological analysis by determining if RDH10 is required by cone photoreceptors for their normal function. If RDH10 is the only enzyme that oxidizes 11-*cis*-retinol as part of the retina visual cycle, its deletion in cone cells should prevent access to this pathway, potentially causing chromophore deficiency and the degeneration of cone cells. Here, we recorded and compared cone responses from *Hrgp-Cre* control mice and *Hrgp-Cre Rdh10* ^*flox/flox*^ mice, in which the expression of *Rdh10* should be abrogated in cone cells. To isolate the cone component of the response, all recordings for this and subsequent cone experiments were performed with mice lacking the rod transducin α subunit (GNAT1). Removal of Gnat1 in mice (*Gnat1* ^−/−^) prevents rods from generating a light response, leaving only the cone-driven response, while preserving the morphology of the retina^32^.

**Figure 2:**
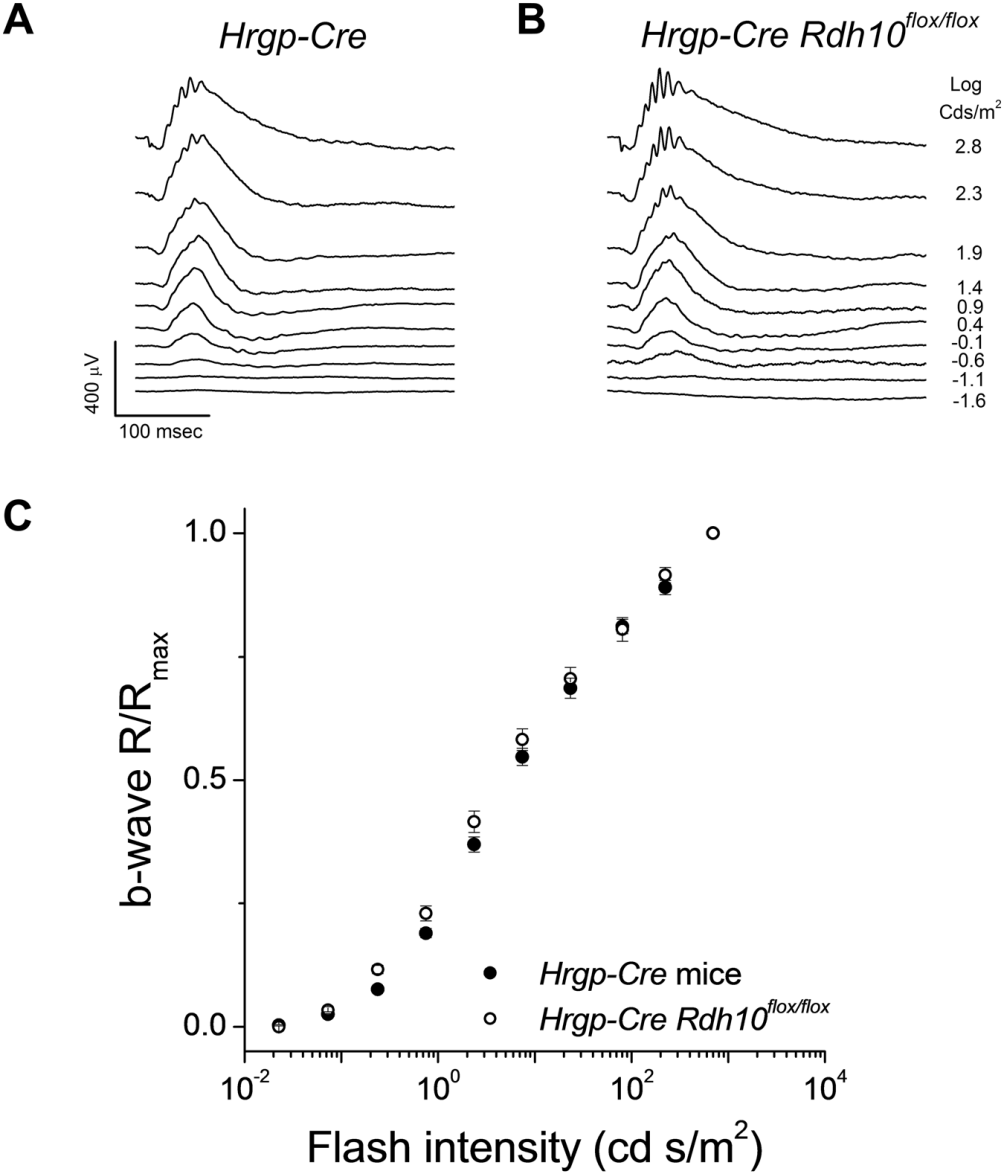
Deletion of RDH10 in cones does not affect the photopic ERG b-wave responses in mice. Representative *in vivo* ERG responses from (**A**) *Hrgp-Cre* control, and (**B**) cone *Rdh10* knockout (*Hrgp-Cre Rdh10* ^*flox/flox*^) mice. (**C**) Ensemble-averaged normalized cone b-wave responses of *Hrgp-Cre* control mice (n = 18, filled symbols) and *Hrgp-Cre Rdh10* ^*flox/flox*^ mice (n = 18, open symbols) as a function of flash intensity. All mice were in *Gnat1* ^−/−^ background to facilitate cone recordings.

Electroretinographic (ERG) recordings *in vivo* revealed comparable cone b-wave maximal response amplitudes between control mice (Fig. 2A) and mice with RDH10-deficient cones (Fig. 2B). This result suggests that the deletion of RDH10 in cones does not affect the overall number of cone cells or their function. The sensitivity of cone-driven b-wave responses also was unaffected by the targeted removal of RDH10 in cones, as their intensity-response curve was comparable to that of control *Hrgp-Cre* mice (Fig. 2C). Together, these findings suggest that cone b-wave responses were unaffected by the deletion of RDH10 in cone cells.

**Figure 3:**
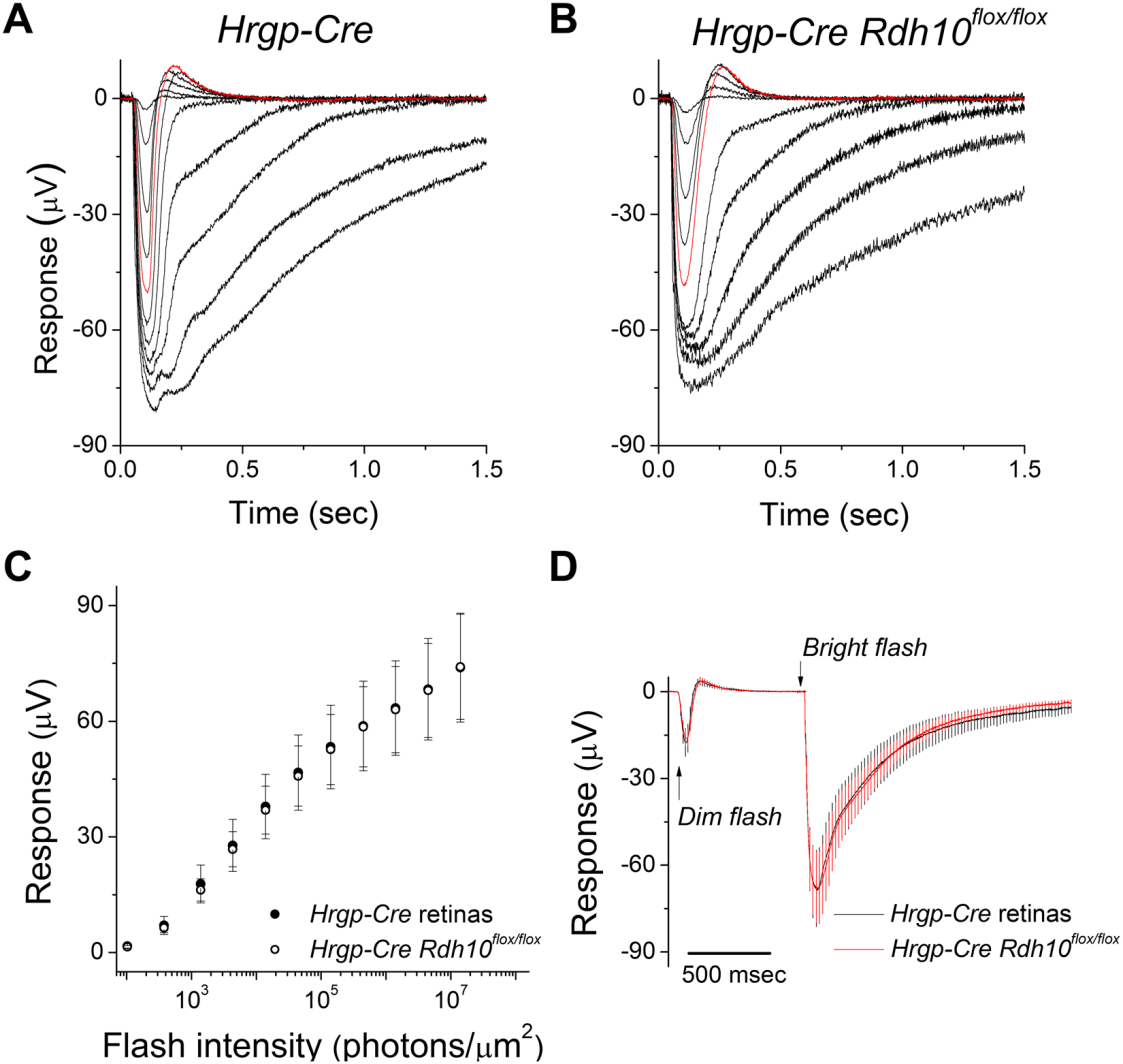
Deletion of RDH10 in cone cells does not affect cone responses. Representative transretinal cone responses from (**A**) *Hrgp-Cre* control retinas, and (**B**) *Hrgp-Cre Rdh10* ^*flox/flox*^ retinas. Red traces: responses to a 1.4 × 10^4^ photons/μm^2^ test flash. (**C**) Ensemble-averaged intensity-response curves of *Hrgp-Cre* control cones (n = 12, filled symbols) and *Hrgp-Cre Rdh10* ^*flox/flox*^ cones (n=12, open symbols). (**D**) Ensemble-averaged dim flash responses (flash intensity: 1.4 × 10^3^ photons/μm^2^) and bright flash responses (flash intensity: 4.5 × 10^6^ photons/μm^2^) of *Hrgp-Cre* control cones (n = 12, black trace) and *Hrgp-Cre Rdh10* ^*flox/flox*^ cones (n = 12, red trace). All mice were in *Gnat1* ^−/−^ background to facilitate cone recordings.

As ERG b-waves represents only an indirect measure of cone photoreceptor function, we also measured directly the cone responses by *ex vivo* transretinal recordings. To isolate the cone-driven a-wave component of the transretinal signal, synaptic transmission was blocked by adding the mGluR6 agonist DL-AP4 to the perfusion solution^33, 34^. The so obtained cone responses from control *Hrgp-Cre* (Fig. 3A) and *Hrgp-Cre Rdh10* ^*flox/flox*^ (Fig. 3B) retinas were similar. Thus, the maximal amplitudes and the sensitivities of the photoresponses were unaffected by the deletion of RDH10 in cone cells (Fig. 3C). Furthermore, the waveforms of the photoresponses to both dim and bright saturating flashes in RDH10-deficient cones were comparable to those in control mice (Fig. 3D). These observations demonstrate that the phototransduction cascade and flash responses in dark-adapted cones are unaffected by the cone-specific deletion of RDH10.

### Cone RDH10 is not required for normal cone dark adaptation

**Figure 4:**
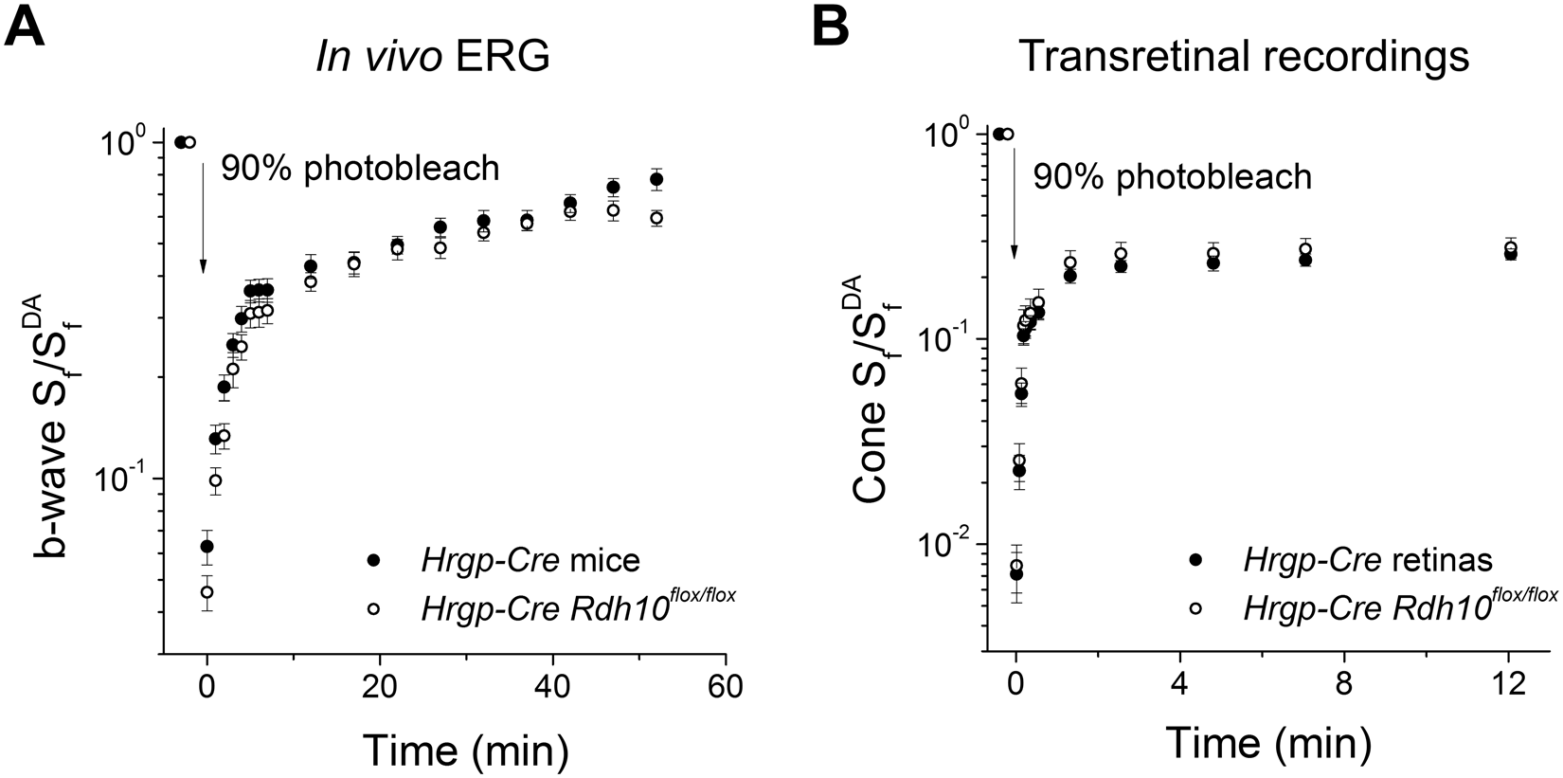
Deletion of RDH10 in cones does not affect cone dark adaptation. (**A**) Normalized cone b-wave sensitivity (b-wave S_f_/b-wave S_f_^DA^) recovery following 90% pigment photobleach from *HrgpP-Cre* control (n = 18, filled symbols) and *Hrgp-Cre Rdh10* ^*flox/flox*^ (n = 18, open symbols) mice measured by *in vivo* ERG recordings. (**B**) Normalized cone sensitivity (S_f_/S_f_^DA^) recovery following 90% pigment photobleach in *Hrgp-Cre* control (n = 13, filled symbols) and *Hrgp-Cre Rdh10* ^*flox/flox*^ (n = 13, open symbols) isolated retinas measured by transretinal recordings. All mice were in *Gnat1* ^−/−^ background to facilitate cone recordings.

Cone dark adaptation *in vivo* is controlled by the combined action of the RPE visual cycle and the Müller cell-dependent retina visual cycle, whereas cone dark adaptation in the isolated retina is driven exclusively by the retina visual cycle^17^. If RDH10 is required for the oxidation of 11-*cis*-retinol in cones, its deletion in these cells should block their access to the retina visual cycle. Therefore, following a strong bleach, RDH10-deficient cones should display a slower overall dark adaptation *in vivo* and should not recover their sensitivity in the isolated retina. To test this hypothesis, we examined whether overall cone dark adaptation *in vivo*, driven by both the RPE and intraretinal visual cycles, is affected by the deletion of *Rdh10* in cones. Initially, cone b-wave sensitivity was estimated under dark-adapted conditions by measuring the amplitudes of dim flash b-wave responses from ERG recordings. Mice were then exposed to bright light to bleach their visual pigment, and cone b-wave sensitivity was measured at set time points during the subsequent dark adaptation. Under these conditions, we found comparable kinetics and final levels of cone b-wave sensitivity recovery in control *Hrgp-Cre* mice and *Hrgp-Cre Rdh10* ^*flox/flox*^ mice (Fig. 4A), suggesting that cone dark adaptation *in vivo* was unaffected by cone-specific deletion of RDH10.

Next, to examine directly the role of cone RDH10 in controlling access to the retina visual cycle, we tested the recovery of sensitivity of RDH10-deficient cones in the isolated retina following exposure to bright light that bleached 90% of the cone pigment. Cone sensitivity was estimated from the cone photoresponses obtained with transretinal recordings, first under dark-adapted conditions and then during cone recovery following exposure of the retina to bright light. Comparison of the kinetics of cone sensitivity recovery following the bleach revealed that dark adaptation in the isolated retina was normal in RDH10-deficient cones (Fig. 4B). Together, these results demonstrate that the ability of cones to access the retina visual cycle, and the efficiency of this pathway, are unaffected by the targeted deletion of RDH10 in cone cells.

### Retina RDH10 is not required for normal dark-adapted cone function

The findings described above demonstrate that RDH10 in cones is not required for normal cone function and dark adaptation. However, a caveat of these experiments is that our immunocytochemical analysis could not establish firmly the expression of RDH10 in cones, whether due to crossreactivity of the RDH10 antibody with Kinectin1 or to the overlap between the processes of Müller cells and cones. In the RPE visual cycle, RDH10 expressed in RPE cells can facilitate 11-*cis*-retinol to 11-*cis*-retinal conversion^22, 24^, and its targeted deletion slows the RPE visual cycle and rod dark adaptation^26^. In the retina, RDH10 is abundantly expressed in the Müller cells^23^. However, its function there and its possible involvement in the retina visual cycle have not been examined. Therefore, to investigate further the role of this enzyme in the retina visual cycle, we generated Müller cell-specific and whole retina-specific conditional RDH10 knockout mice by crossing *Rdh10* ^*flox/flox*^ mice with the *Pdgfra-Cre* line and the *Six3-Cre* line, respectively.

**Figure 5:**
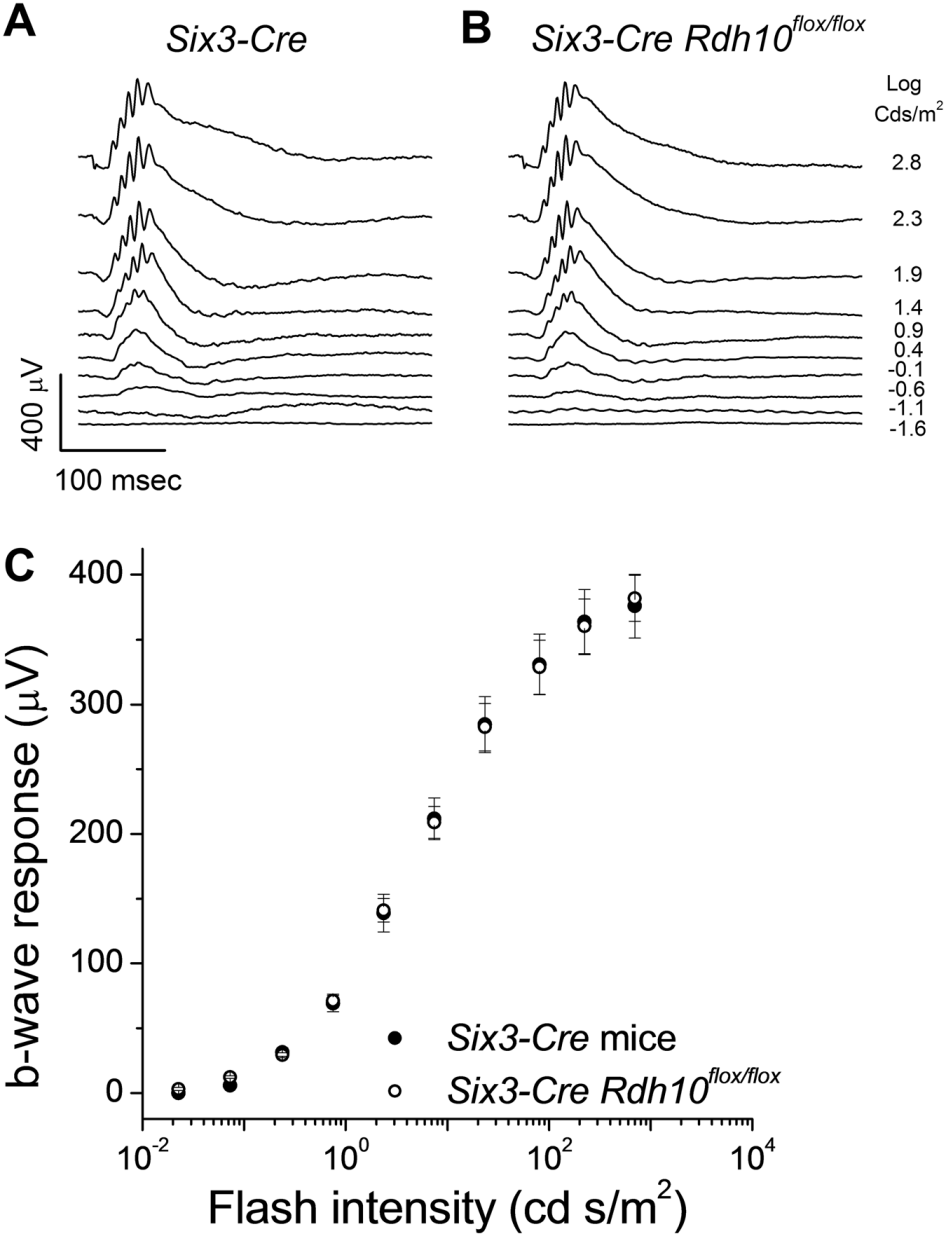
Deletion of RDH10 in the entire retina does not affect the photopic ERG b-wave responses in mice. Representative *in vivo* ERG responses of (**A**) *Six3-Cre* control and (**B**) retina *Rdh10* knockout (*Six3-Cre Rdh10* ^*flox/flox*^) mice. (**C**) Ensemble-averaged cone b-wave intensity-response curves of *Six3-Cre* control mice (n = 12, filled symbols) and *Six3-Cre Rdh10* ^*flox/flox*^ mice (n = 10, open symbols). All mice were in *Gnat1* ^−/−^ background to facilitate cone recordings.

**Figure 6:**
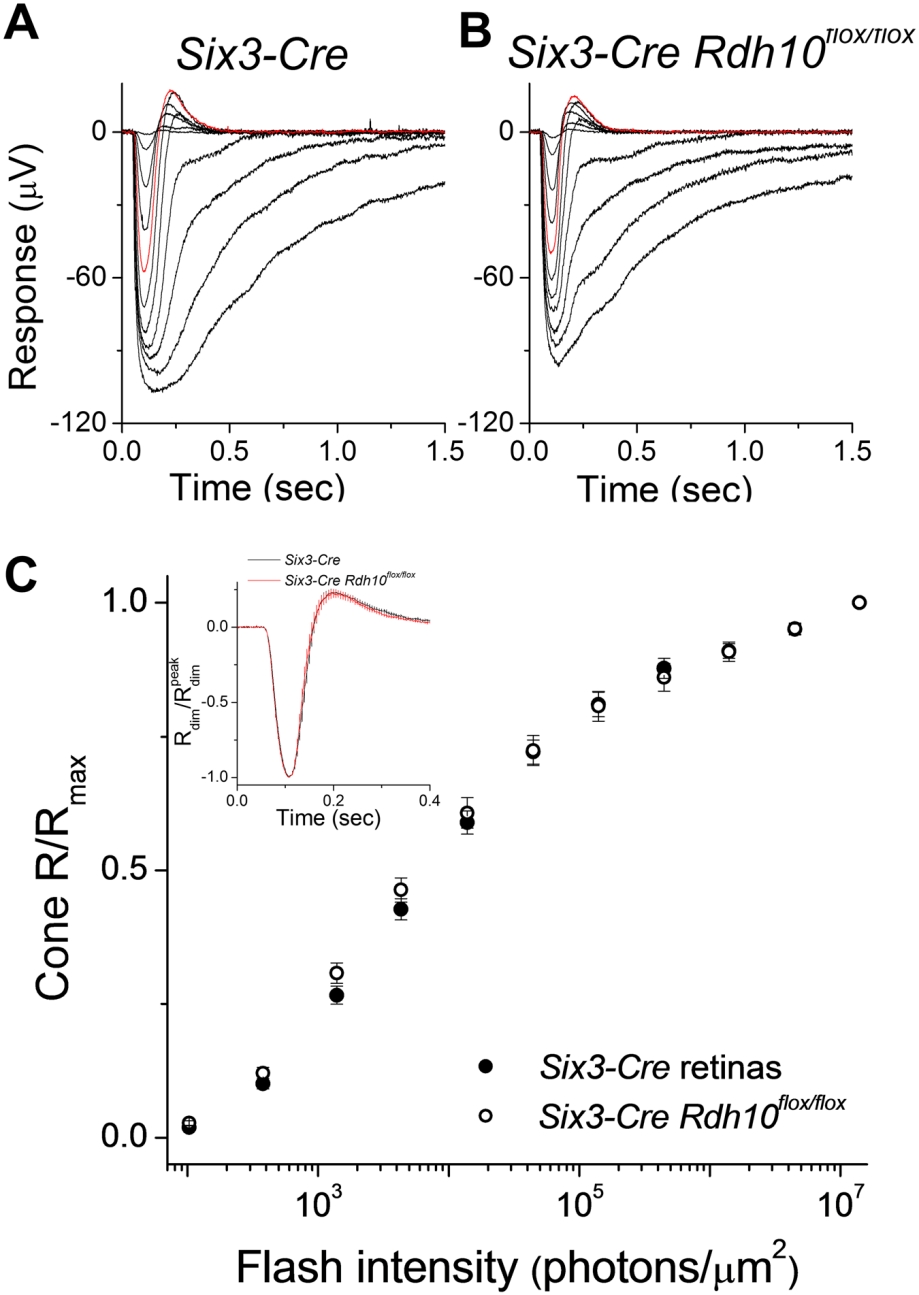
Deletion of RDH10 in the entire retina does not affect cone responses. Representative transretinal cone responses from (**A**) *Six3-Cre* control, and (**B**) *Six3-Cre Rdh10* ^*flox/flox*^ retinas. Red traces: responses to a 1.4 × 10^4^ photons/μm^2^ test flash. (**C**) Normalized cone intensity-response curves of *Six3-Cre* control (n = 9, filled symbols) and *Six3-Cre Rdh10* ^*flox/flox*^ (n = 8, open symbols) retinas. Inset: normalized cone dim flash responses from *Six3-Cre* control (n = 9, black trace) and *Six3-Cre Rdh10* ^*flox/flox*^ (n = 8, red trace) retinas. All mice were in *Gnat1* ^−/−^ background to facilitate cone recordings.

*In vivo* ERG recordings demonstrated that the cone-driven b-wave responses in mice lacking RDH10 selectively in Müller cells (not shown) and in mice lacking RDH10 in the entire retina display normal dark-adapted response waveforms (Fig. 5A and B), response amplitudes, and sensitivity (Fig. 5C). A more detailed examination of cone function by *ex vivo* transretinal recordings confirmed that cone function was unaffected by the deletion of RDH10 in the retina. Maximal response amplitudes (Fig. 6A and B), the response waveforms (Fig. 6C, inset), and cone sensitivity as measured from the intensity-response curve (Fig. 6C) were all normal in *Six3-Cre Rdh10* ^*flox/flox*^ mice as compared to *Six3-Cre* controls. Thus, the function of cones under dark-adapted conditions appeared unaffected by deletion of RDH10 in either Müller cells or in the entire retina.

### Retina RDH10 is not required for cone dark adaptation and the retina visual cycle

**Figure 7:**
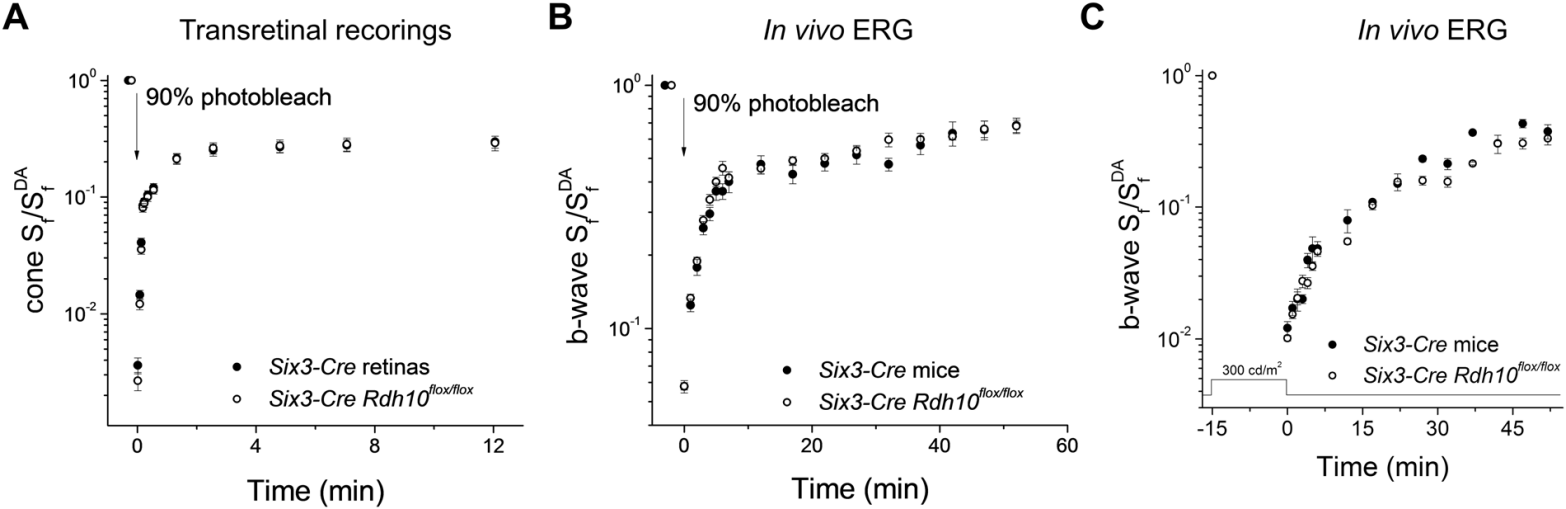
Deletion of RDH10 in the entire retina does not affect cone dark adaptation. (**A**) Normalized cone sensitivity (S_f_/S_f_^DA^) recovery following 90% pigment photobleach of *Six3-Cre* control (n = 9, filled symbols) and *Six3-Cre Rdh10* ^*flox/flox*^ (n = 8, open symbols) retinas measured by transretinal recordings. (**B**) Normalized cone b-wave sensitivity (b-wave S_f_/b-wave S_f_^DA^) recovery following a 90% pigment photobleach of *Six3-Cre* control (n = 12, filled symbols) and *Six3-Cre Rdh10* ^*flox/flox*^ (n = 10, open symbols) mice measured by *in vivo* ERG recordings. (**C**) Normalized cone b-wave sensitivity (b-wave S_f_/b-wave S_f_^DA^) recovery following a 15 min exposure to 300 cd/m^2^ Ganzfeld background light (530 nm wavelength) from *Six3-Cre* control (n = 4, filled symbols) and *Six3-Cre Rdh10* ^*flox/flox*^ (n = 4, open symbols) mice measured by *in vivo* ERG recordings. All mice were in *Gnat1* ^−/−^ background to facilitate cone recordings.

Next, we examined how deletion of RDH10 from the entire retina affects the retina visual cycle and cone dark adaptation. *Ex vivo* transretinal recordings were used to monitor cone dark adaptation driven by the retina visual cycle. The kinetics of cone sensitivity recovery following exposure to bright light in *Six3-Cre Rdh10* ^*flox/flox*^ retinas were comparable to those in control *Six3-Cre* retinas (Fig. 7A). This outcome suggests that RDH10 in Müller glia is not required for the function of the retina visual cycle. Corroborating this finding, cone dark adaptation *in vivo*, driven by the combined action of the retina and RPE visual cycles, also was unaffected by deletion of RDH10 from the retina (Fig. 7B). Due to the lack of phenotype of mice lacking RDH10 in the entire retina, we did not examine the dark adaptation of cones from mice with targeted deletion of RDH10 in Müller cells.

To test the function of the retina visual cycle under extreme conditions of illumination, we exposed mice to 15 min of bright background light to deplete any potential stores of chromophore in the retina and RPE. The subsequent dark adaptation of cones, as measured by the *in vivo* ERG cone b-wave response, proceeded with comparable kinetics in control mice and mice with RDH10-deficient retinas (Fig. 7C). Thus, this experiment revealed no evidence for a role of RDH10 in either cone cells or Müller cells in the retina visual cycle.

### RDH10 is not sufficient for enabling access to the retina visual cycle

**Figure 8:**
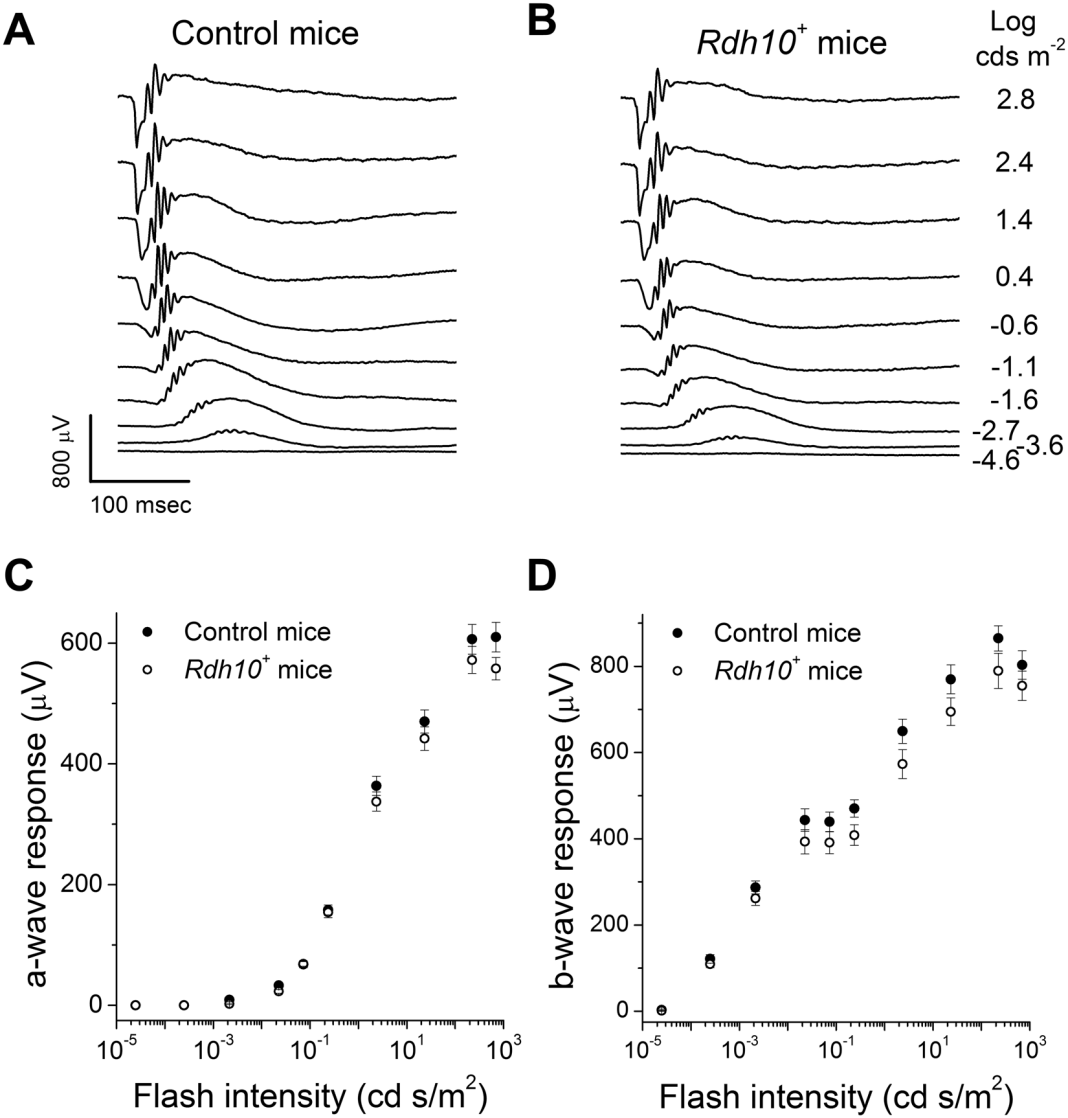
Ectopic expression of RDH10 in rod photoreceptor cells does not affect their photoresponses. Representative scotopic *in vivo* ERG responses from (**A**) control, and (**B**) transgenic *Rdh10* (*Rdh10* ^+^) mice. (**C**) Ensemble-averaged scotopic a-wave responses (**C**) and scotopic b-wave responses (**D**) of control (n = 9, filled symbols) and *Rdh10* ^+^ (n = 9, open symbols) mice measured by *in vivo* ERG recordings.

The functional redundancy of RDH enzymes has been reported in RPE cells and rod photoreceptors^26, 35^. Such redundancy of RDH enzymes in cones and Müller cells could obscure any role of RDH10 based on loss-of-function experiments alone. Thus, we sought to determine the possible role of RDH10 in controlling access to the retina visual cycle by introducing ectopic RDH10 in mouse rods. *In vivo* ERG recordings from *Rdh10* ^+^ transgenic mice revealed normal scotopic responses (compare Fig. 8A and B). Moreover, the intensity-response curves of both the rod-driven a-waves (Fig. 8C) and rod bipolar cell-driven b-waves (Fig. 8D) in *Rdh10* ^+^ mice were comparable to those in wild-type controls.

**Figure 9:**
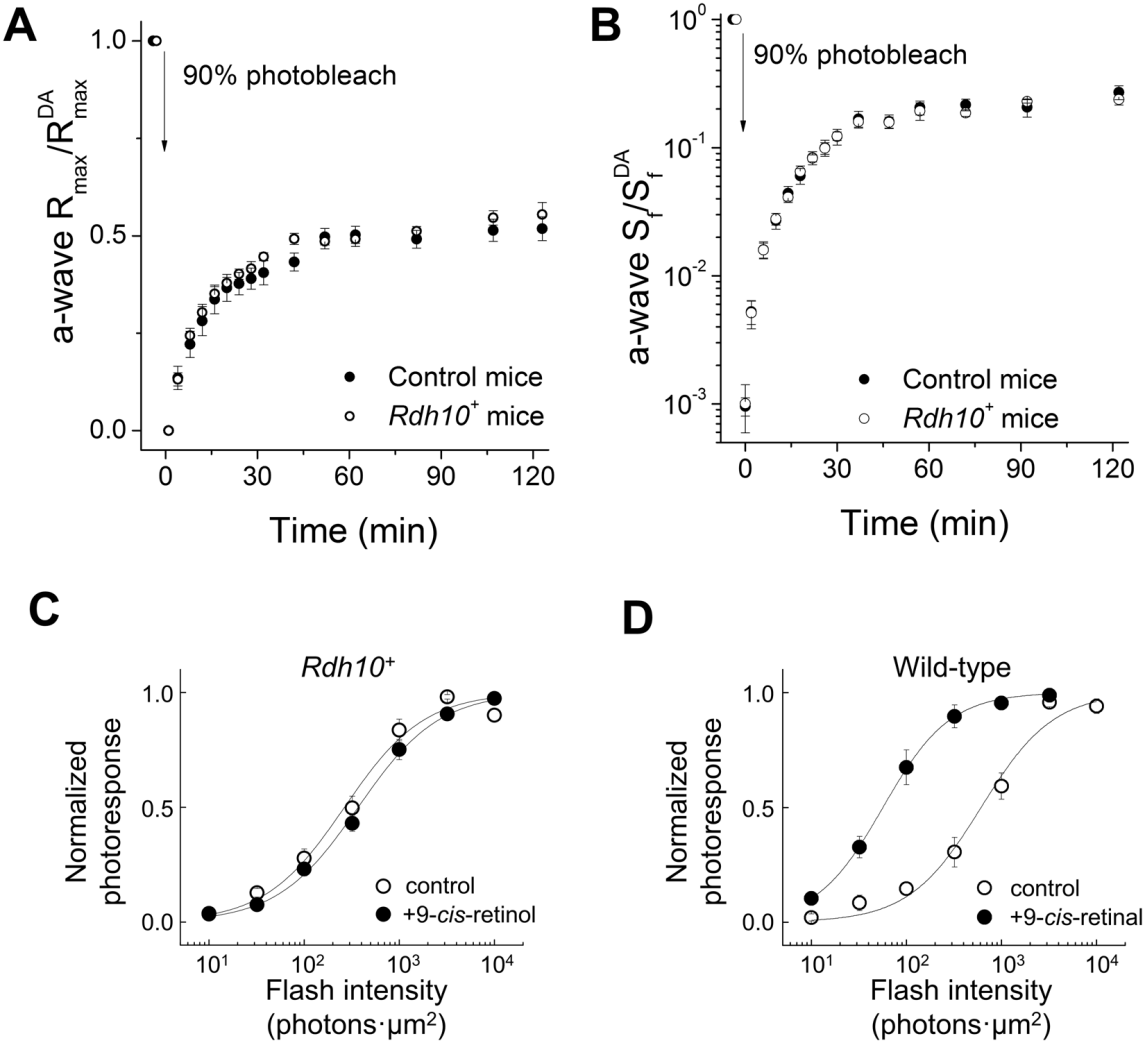
Ectopic expression of RDH10 does not enable rods to dark adapt faster or to oxidize *cis*-retinol for pigment regeneration. (**A** and **B**) Normalized rod scotopic a-wave maximal response (R_max_/R_max_^DA^; **A**) or sensitivity (S_f_/S_f_^DA^; **B**) recovery following a 90% pigment photobleach in control (n = 9, filled symbols) and *Rdh10* ^+^ (n = 9, open symbols) mice measured by *in vivo* ERG recordings. (**C** and **D**) Normalized intensity-response curves determined by single-cell suction recordings from (**C**) transgenic *Rdh10* ^+^ rods incubated in control solution (n = 13, open symbols) or in 100 µM 9-*cis*-retinol solution (n = 14, filled symbols), or from (**D**) wild-type rods incubated in control solution (n = 6, open symbols) or in 100 µM 9-*cis*-retinal solution (n = 11, filled symbols) following 50% pigment photobleach.

If RDH10 expressed in rod cells enables them to access the retina visual cycle, their dark adaptation following a bleach should be accelerated, as in hybrid *rd7* rods^5^. However, the kinetics of recovery of both the a-wave maximal amplitude (Fig. 9A) and a-wave sensitivity (Fig. 9B) in *Rdh10* ^+^ mice determined by *in vivo* ERG recordings were comparable to those measured in wild-type controls. One potential caveat to interpreting this experiment is that structural differences between rods and cones might also be involved in controlling access to the retina visual cycle^36^. For example, the lack of acceleration in rod recovery could be due to the inability of Müller cells to supply retinoid to rod cells. Therefore, to directly test the ability of *Rdh10* ^+^ rods to oxidize *cis*-retinol, we treated bleached control and *Rdh10* ^+^ retinas with exogenous retinoid, thereby bypassing the Müller cell-mediated delivery of retinoids. We used the more stable and commercially-available 9-*cis*-analog of 11-*cis*-retinol, which can be readily oxidized by amphibian^15^ and mouse^37^ cones and used for pigment regeneration. However, treatment of *Rdh10* ^+^ rods with 9-*cis*-retinol did not promote a recovery in their sensitivity (Fig. 9C), indicating a lack of pigment regeneration. In contrast, 9-*cis*-retinal used with the same protocol promoted robust recovery in wild-type rods (Fig. 9D). Together, these results demonstrate that the expression of RDH10 in rod cells is insufficient for them to oxidize *cis-*retinol or to access the retina visual cycle.

## Discussion

It has long been known that cone cells dark adapt faster than rod cells^38^. In addition to the faster decay and regeneration of cone visual pigments compared to rhodopsin^39^, such rapid dark adaptation could be facilitated by the ability of cone cells to oxidize 11-*cis*-retinol produced by Müller cells^9, 16^. Recently, we identified cellular retinaldehyde binding protein (CRALBP) in Müller cells as the first functionally confirmed molecular component of the retina visual cycle^20^. However, the rest of this molecular pathway remains uncharacterized. RDH10 expressed in RPE and Müller cells has been proposed to support the visual cycle^23, 24^. A recent report demonstrated that the deletion of RDH10 selectively in RPE cells indeed compromised rod function^26^. Here, we examined the possible role of RDH10 as the cone-specific 11-*cis*-RDH. Surprisingly, considering the robust expression of RDH10 in the retina, its targeted deletion in cones, Müller cells, or the entire retina did not affect the overall viability and visual function of cone cells, and, critically, did not suppress their dark adaptation. These findings suggest that RDH10 is not involved in the recycling of chromophore in the retina and does not play a role in cone pigment regeneration. Alternatively, it is possible that redundancy in retina RDHs, similar to the case in rod photoreceptors^35^ and RPE cells^26^ prevented us from observing a cone phenotype in the retinas of RDH10-deficient mice. Indeed, RDH10 and another RDH, retSDR1, have been reported to activate each other in a reciprocal manner to maintain retinoid homeostasis^40^.

Our study presents the first attempt to identify the 11-*cis*-RDH(s) in mammalian cone cells and to determine the function of RDH10 in the retina visual cycle. Because the deletion of RDH10 in the entire retina, including Müller cells and cones, in the current study did not affect cone function and dark adaptation, it is unlikely that RDH10 enables Müller cells to produce 11-*cis*-retinal for use by cones or enables cones to oxidize 11-*cis*-retinol produced by Müller cells. While the function of RDH10 in the adult retina remains unclear, it has been suggested that it might support retinal development by producing the retinaldehyde substrate for retinoic acid biosynthesis^28, 41^.

Transgenic *Rdh10* ^+^ rod cells did not demonstrate an acceleration in their dark adaptation, and rod-driven *in vivo* ERG responses remained normal, suggesting that unlike *rd7* rods, these transgenic rods were unable to use 11-*cis*-retinol produced by the retina visual cycle. To rule out restrictions in the flow of retinoids between *Rdh10* ^+^ rods and the processes of Müller cells^36^, we also directly tested the ability of rod cells expressing RDH10 to utilize *cis*-retinol for pigment regeneration. However, we observed no evidence of pigment regeneration. These findings clearly demonstrate that RDH10 is insufficient for *cis*-retinol oxidation and access to the retina visual cycle.

Our extensive results demonstrating that RDH10 is not required for the function of the retina visual cycle leave open the question of the identity of the *cis*-retinol oxidation enzyme in cone cells. Are there other candidates for the 11-*cis*-RDH? A recent study demonstrated that in the salamander, oxidation of *cis*-retinol occurs in the outer segments of cones^42^. Therefore, the cone-specific *cis* retinol RDH would be expected to be localized in the outer segments of cones. As reviewed by Parker and Crouch, both retSDR1 and RDH8 are found in cone outer segments, whereas RDH12 is in cone inner segments^43^. A more recent review of RDHs^44^ includes information on RDH13, expressed in human, monkey, and mouse rod and cone inner segments^45^ and RDH14, localized in the outer segments of bovine rods and cones, as well as in Müller cells^46^. The cell specificity of RDH14 in mouse retina and its subcellular distribution have not been determined. Considering that RDH8, RDH12, and RDH13 are also expressed in rod cells, they are unlikely to be the cone-specific RDH that mediates access to the retina visual cycle, leaving retSDR1 and RDH14 as potential candidates. Based on a recent biochemical study of carp cones, RDH13L also has been proposed to be the essential 11-*cis-*RDH^47^, suggesting a similar role for its mammalian homolog, RDH14^48^. The physiological role of RDH14 has not been examined to date and therefore, future studies should focus on evaluating RDH13L in fish and RDH14 in mammals as likely *cis*-retinol oxidation enzymes of the cone-specific retina visual cycle. The functional role of retSDR1, which reciprocally modulates RDH10, also remains to be established in cone photoreceptors.

## Methods

### Animals

All experimental protocols were in accordance with the Guide for the Care and Use of Laboratory Animals and were approved by the Washington University Animal Studies Committee and the Case Western Reserve University Animal Care Committee. Unless otherwise specified, experiments were performed with 2-4 month-old mice of both sexes using their littermates or age-matched animals as controls. The cone-specific Cre mice, *Hrgp-Cre* ^29^, the Müller cell-specific Cre mice, *Pdgfra-Cre* ^30^, and the retina-specific Cre mice, *Six3-Cre* ^31^ were obtained from Jackson Laboratory (Bar Harbor, ME). *Rdh10* ^*flox/flox*^ mice^28^ were crossed with *Hrgp-Cre* mice to delete RDH10 selectively in cone cells. Müller cell-specific RDH10 knockout mice were generated by crossing *Rdh10* ^*flox/flox*^ mice with *Pdgfra-Cre* mice. Finally, *Rdh10* ^*flox/flox*^ mice were crossed with *Six3-Cre* mice to delete RDH10 in the entire retina. The cone-specific Cre and retina-specific Cre experiments were performed with mice lacking the rod transducin α subunit (*Gnat1* ^−/−^ mice kindly provided by Dr. Janis Lem, Tufts University, Boston), which greatly facilitated the isolation of the cone component of the light response without compromising retinal morphology^32^.

Transgenic *Rdh10* mice (*Rdh10* ^+^) were generated by the Transgenic Core in the Department of Ophthalmology and Visual Sciences at Washington University. The transgene of the mouse *Rdh10* cDNA was introduced by the human rhodopsin kinase promoter^49^ in order to drive RDH10 expression ectopically in rod photoreceptors. The mouse *Rdh10* sequence was described in a previous study^22^.

### Immunoblotting analyses

The RDH10 antibody was obtained from ProteinTech, Rosemont, IL (#14644). Both retinas from each mouse were collected in 200 µl 2X Laemmli buffer (2% (w/v) SDS, 62.5 mM Tris-HCl pH 6.8, 25% (w/v) glycerol, 0.04% (w/v) bromophenol blue) supplemented with 5% (v/v) β-mercaptoethanol and mechanically disrupted with zirconium beads (Next Advance, Averill Park, NY) for 3 min at speed 6. Homogenates were then boiled and centrifuged for 10 min at 17,000 *g*. Thirty microliters of each supernatant were used per well for immunoblotting analyses.

### Electrophysiology

*In vivo* electroretinography (ERG) and *ex vivo* transretinal recordings were performed to measure photoreceptor function and dark adaptation as previously described^20, 50^. Prior to these tests, mice were transferred to a light-proof cabinet with water and food and dark-adapted for at least 18 h. For *in vivo* ERG experiments, dark-adapted mice were anesthetized by intraperitoneal injection of 100/20 mg/kg ketamine/xylazine cocktail and placed on a heating pad with thermal feedback to avoid hypothermia. Eyes were treated with 1% (w/v) atropine sulfate and 2.5% (w/v) hypromellose solution (Akorn Pharmaceuticals, Lake Forest, IL) to dilate the pupils and ensure proper contact with the electrodes. Contact-lens electrodes were then carefully attached to the cornea for recording the transretinal voltage signals relative to a needle electrode inserted under the scalp. After stabilizing the baseline in darkness for 15 min, ERG light responses were triggered and recorded by using a UTAS Visual Diagnostic System with BigShot^TM^ Ganzfeld (LKC Technologies, Gaithersburg, MD). Flash intensities of the Ganzfeld LED were calibrated with an S471 optometer (UDT Instruments, San Diego, CA). To maximize the photoactivation of mouse rhodopsin (502 nm) and M/L cone visual pigment (508 nm)^51^, 530 nm wavelength Ganzfeld flashes were used for light stimuli below 25 cds/m^2^ and white xenon flashes were used for flash intensities higher than 25 cds/m^2^. Multiple sweeps per flash intensity were averaged and analyzed with a custom-programmed Excel document.

For transretinal recordings, dark-adapted mice were euthanized with CO_2_ followed by cervical dislocation. Eyes were immediately enucleated and the corneas and lenses removed. Retinas were dissected from the rest of the eye and placed in a Petri dish filled with Locke’s solution, mounted into a custom-made recording chamber^52^, and perfused with 33 °C Locke’s solution, containing 0.1% minimum essential media (MEM) vitamins and 0.1% MEM amino acids (MilliporeSigma, St. Louis, MO) and bubbled with O_2_/CO_2_ (95%/5%). Retinas were perfused for 15 min before the recordings to allow signal stabilization. Test flashes of 512 nm wavelength to selectively excite M/L cone visual pigment were generated with a custom-built LED system. Retinal flash responses were amplified by a differential amplifier (DP-311; Warner Instruments, Hamden, CT), low-pass filtered at 30 Hz by an 8-pole Bessel filter (3382; Krohn-Hite Corporation, Brockton, MA), digitized at 1 kHz (Axon Digidata 1440A, Molecular Devices, Sunnyvale, CA), and then recorded and analyzed with Axon pCLAMP software (Molecular Devices). The results of all electrophysiological experiments were graphed with Origin (OriginLab, Northampton, MA).

For dark adaptation experiments, eyes or dissected retinas were briefly exposed to bright green LED light (30 seconds of 505 nm for *in vivo* ERGs and 3 seconds of 512 nm for *ex vivo* transretinal recordings to achieve ~90% photobleach), immediately followed by a series of timed test flashes to estimate the sensitivity (S_f_) and/or maximal response amplitude (R_max_) of photoreceptors as previously described^20, 50^. The sensitivity and maximal response amplitude values were then normalized to their corresponding pre-bleached dark-adapted levels (S_f_^DA^ and R_max_^DA^). To explore the efficiency of the visual cycle of RDH10-null retinas during and after a prolonged photobleach, *Six3-Cre Rdh10* ^*flox/flox*^ mice were also exposed to 15 min of 300 cd/m^2^ 530 nm light before recording their recovery as previously described^37^.

Sensitivity recovery measurements from rod photoreceptors were performed as follows. First, retinas obtained from dark-adapted mice were transferred to a Petri dish filled with Locke’s solution supplemented with 0.1% (w/v) lipid-free bovine serum albumin (A6003, MilliporeSigma, Saint Louis, MO) to promote the release of toxic all-*trans*-retinal produced after the photobleach. Retinas then were exposed to light (500 nm, 1.0 × 10^6^ photons/µm^2^) for 2 min to photobleach 50% of the rhodopsin. Bleached retinas were immediately transferred to another dish containing Locke’s solution supplemented with 100 µM 9-*cis*-retinol (Santa Cruz Biotechnology, Inc., Santa Cruz, CA) or 9-*cis*-retinal (MilliporeSigma), 0.1% (v/v) ethanol and 1% (w/v) lipid-free bovine serum albumin (to promote the delivery of hydrophobic retinoids to rods) and incubated in an oxygenated dark box at room temperature for 3 h. Finally, single-cell suction recordings were obtained from rods as previously described^5^.

*RNA-seq*. Retinas were harvested from 3-week-old C57BL/6J and *rd7* mice. For each strain, two biological replicates, each consisting of 8 retinas from two males and two females, were collected and processed as previously described^53^. The library was subjected to one lane of sequencing on the Illumina HiSeq 2000 (single 50 base pair reads) at the Genome Technology Access Center at Washington University School of Medicine. Reads were mapped to mm9 using TopHat v1.4.1 and analyzed with Cufflinks v1.3.0 for transcript assembly and differential expression^54, 55^. A total of 1.9 × 10^8^ reads were generated, of which 1.7 × 10^8^ were mapped, and biological replicates showed a high degree of correlation (r^2^ > 0.97). Genes were identified as significantly differentially expressed at a false discovery rate (FDR) of 5%.

*qRT-PCR*. Retinas were dissected from mice and total RNA was isolated with the RNeasy Mini kit (Qiagen, Valencia, CA). Total cDNA was synthesized with a quantitative cDNA Kit (Qiagen). qRT-PCR was performed with SYBR Green Master (Roche Applied Science, Basel, Switzerland). Gene expression was normalized with Gapdh.

### Data Access

Raw and processed RNA-seq data have been deposited in Gene Expression Omnibus (GEO; accession number GSE86442).

## Electronic supplementary material

Supplementary Information

Supplemental Table 1
