# Supplementary material for: The role of retinol dehydrogenase 10 in the cone visual cycle
Source: Sci Rep. 2017 May 24;7:2390. doi: 10.1038/s41598-017-02549-8 (PMC5443843; doi:10.1038/s41598-017-02549-8)
Supplement: Supplementary file 1 — Supplementary Information [file 41598_2017_2549_MOESM1_ESM.pdf]

The role of retinol dehydrogenase 10 in the cone visual cycle

Yunlu Xue[1,#a], Shinya Sato[1], David Razafsky[1,#b], Bhubanananda Sahu[2], Susan Q. Shen[3], Chloe Potter[1], Lisa L. Sandell[4], Joseph C. Corbo[3], Krzysztof Palczewski[5], Akiko Maeda[2,5], Didier Hodzic[1], and Vladimir J. Kefalov[1*]

Supplemental Table 1. Significantly differentially expressed genes in *rd7* vs. wild-type retinas by RNA-seq.
